# Knowledge, Awareness, Attitudes, Acceptance, and Uptake of the Herpes Zoster Vaccine in Saudi Arabia: A Scoping Review

**DOI:** 10.3390/vaccines14070565

**Published:** 2026-06-26

**Authors:** Howeida Abusalih

**Affiliations:** Department of Health Sciences, College of Health and Rehabilitation Sciences, Princess Nourah Bint Abdulrahman University, P.O. Box 84428, Riyadh 11671, Saudi Arabia; hhabusalih@pnu.edu.sa

**Keywords:** herpes zoster, shingles, vaccine hesitancy, primary healthcare, knowledge, attitudes, Saudi Arabia, awareness, acceptance, uptake

## Abstract

**Background**: Herpes zoster (HZ), commonly known as shingles, and post-herpetic neuralgia (PHN) represent growing public health concerns, particularly among older adults. Despite the established efficacy of the herpes zoster vaccine (HZV), global uptake remains suboptimal. **Objectives**: This scoping review maps evidence from Saudi Arabia evaluating the baseline knowledge, awareness, attitudes, acceptance, hesitancy, and clinical uptake of the HZV among general adults, high-risk populations, and healthcare workers (HCWs). **Methods**: The JBI and PRISMA-ScR methodological frameworks were strictly adhered to during mapping. Eligible sources included peer-reviewed, observational cross-sectional studies conducted in Saudi Arabia and published in English between 2022 and 2026. The search was conducted across PubMed, Scopus, Web of Science, and Google Scholar. Data were systematically extracted and charted using a standardized digital piloting framework to capture study characteristics (author, year, and region), sample sizes, target populations, knowledge percentages, actual vaccine uptake rates, and self-reported barriers. **Results**: Out of 25 retrieved records, 19 unique primary studies were mapped. Public knowledge of HZ complications and vaccine eligibility criteria was consistently low to moderate, falling below 50% across most cohorts. Conversely, while verbal willingness to receive the vaccine was highly favorable (ranging from 60% to 75%), a profound “intention–behavior gap” was observed, with actual clinical uptake being below 10%. Key barriers included a lack of public health campaigns, safety concerns regarding reactogenicity, online misinformation, and a lack of proactive provider communication. For HCWs, barriers included unclear local guidelines and a lack of workplace mandates. Ultimately, a proactive physician recommendation was identified as the single most powerful clinical facilitator, increasing vaccine acceptance by over 80% across all cohorts. **Conclusions**: While the shingles vaccine is now distributed completely free across Saudi Arabia, high public willingness has not translated into actual vaccination rates (10%) due to low public awareness of disease severity. Free vaccine availability alone is insufficient; primary care systems must shift from a passive delivery model to an active, provider-driven framework to successfully close this gap

## 1. Introduction

Herpes zoster (HZ), commonly characterized as shingles, represents a substantial global public health burden, primarily threatening older adults and immunocompromised individuals due to age-related cellular immune decline [1,2]. Despite the availability of highly effective clinical interventions, preventive immunization strategies are increasingly stymied by complex behavioral roadblocks [2]. Historically, the World Health Organization characterized vaccine hesitancy as a critical threat to global health security [3]. Vaccine hesitancy operates as a universal, multi-faceted phenomenon driven by a complex matrix of social, cultural, and psychological variables that shape individual risk perception and healthcare encounters globally [4].

This epidemiological challenge is acutely mirrored within the Kingdom of Saudi Arabia (KSA), where demographic shifts toward an aging population paired with an increasing prevalence of chronic comorbid conditions have elevated the clinical relevance of HZ [5]. Evidence from regional primary healthcare settings indicates that HZ significantly impacts adult populations, with post-herpetic neuralgia (PHN) manifesting as a debilitating, long-term neuropathic complication that severely erodes patient quality of life [5,6]. National surveillance metrics confirm that individuals aged 50 years and older experience the highest rates of acute HZ manifestations and secondary neuralgic sequelae [6,7].

To mitigate this clinical and socioeconomic burden, the Saudi Ministry of Health formally introduced the recombinant zoster vaccine (HZV) into national immunization channels on 29 September 2022 [8]. However, widespread institutional availability has not automatically translated into optimal public health outcomes, as baseline public awareness and empirical vaccine deployment remain highly variable across different provinces [9]. While preliminary cross-sectional investigations have emerged across various metropolitan and suburban hubs to assess public comprehension of HZ etiology and vaccine efficacy, the available literature exhibits notable fragmentation regarding how knowledge deficits vary across diverse demographics [10,11].

“Furthermore, behavioral dynamics within the Kingdom present a stark paradox: while public attitudes toward the HZV are generally characterized as positive, actual real-world clinical uptake remains heavily depressed [12,13]. This pronounced intention–behavior gap points to critical systemic bottlenecks, including a historical reliance on passive patient intent rather than active, provider-initiated clinical pathways [9]. Emerging research suggests that proactive physician recommendations may act as a primary determinant of vaccine acceptance, yet substantial gaps persist regarding how healthcare workers (HCWs) independently execute vaccine advocacy [14]. Frontline medical professionals face distinct multi-level barriers—ranging from institutional vaccine stock inconsistencies to a lack of structured continuing medical education modules regarding adult immunizations—which limits their capacity to counter pervasive misinformation networks circulating in regional digital channels [15,16].”

While isolated regional studies have separately explored consumer barriers, access limitations, and localized facilitators, there has been no comprehensive, macro-level synthesis of these disparate findings to date [17,18]. Systematically mapping the specific cognitive, psychological, and structural factors governing HZV dynamics is essential to inform targeted educational frameworks and optimize preventive adult immunization strategies within the Kingdom.

The objective of this scoping review is to systematically map and synthesize the peer-reviewed evidence regarding Herpes Zoster and its vaccine’s awareness, knowledge, attitudes, practices, barriers, and clinical facilitators among the general public, target older adults, high-risk cohorts, and healthcare professionals within the primary care and preventive healthcare framework of the Kingdom of Saudi Arabia.

## 2. Methodology

### 2.1. Methodological Approach

This scoping review was conducted following the Joanna Briggs Institute (JBI) methodological guidance for scoping reviews [19] and reported following the PRISMA-ScR (Preferred Reporting Items extension for Scoping Reviews) checklist, presented in Supplementary File S1 [20]. To ensure originality, methodological rigor, and transparency, the review protocol was registered with the International Platform of Registered Systematic Review and Meta-analysis Protocols (INPLASY202650084; doi: 10.37766/inplasy2026.5.0084).

This review adhered to the five-stage framework proposed by Arksey O’Malley and levac [21,22], incorporating refinements from later methodological developments. The stages included the following:

1. Defining the research question;
2. Identifying relevant literature;
3. Selecting studies;
4. Extracting and organizing the data;
5. Mapping, summarizing, and presenting the findings.

To ensure conceptual clarity and relevance to the target setting and participants, the Population–Concept–Context (PCC) framework was used to guide the formulation of eligibility criteria and the scope of the review. Specifically, the population encompassed adults and healthcare workers, the core concept focused on HZV-related awareness and behaviors, and the context was geographically bounded to KSA.

### 2.2. Search Strategy

A comprehensive and organized electronic literature search strategy (Supplementary File S1) was conducted across major medical, nursing, and public health databases selected for their extensive coverage of epidemiology, vaccination, and regional healthcare research. These included PubMed, Scopus, Web of Science, and Google Scholar. The search targeted peer-reviewed articles published between January 2022 and April 2026 and was limited to English-language studies with full-text availability.

To maximize precision, the search strategy combined controlled vocabulary such as Medical Subject Heading (MeSH) terms with free-text keywords. To ensure relevance, the terms were organized into five distinct thematic categories reflecting the review’s core scope:

- Disease Terms: “Herpes zoster”, “shingles”, “HZ”, “post-herpetic neuralgia”, and “PHN”.
- Vaccine Terms: “Herpes zoster vaccine”, “HZV”, “shingles vaccine”, “zoster immunization”, “vaccine uptake”, “vaccine acceptance”, and “vaccine hesitancy”.
- Population Terms: “Adults”, “older adults”, “elderly”, “healthcare workers”, and “general population”.
- Regional Terms: “Saudi Arabia”, “KSA”, “Riyadh”, “Jeddah”, “Al Ahsa”, and “Makkah”.
- Behavioral/Attitudinal Terms: “Knowledge”, “awareness”, “attitudes”, “perceptions”, “beliefs”, “barriers”, and “facilitators”.

Boolean operators (AND, OR, NOT) and exact phrase searching were applied to refine the results. For a study to be considered eligible, it had to include at least one term or phrase from each of the primary conceptual categories. A representative example of a combined search string used across the databases is as follows:

[“Herpes zoster” OR “shingles”] AND [“vaccine” OR “immunization”] AND [“awareness” OR “acceptance” OR “hesitancy”] AND [“Saudi Arabia” OR “KSA” OR “Riyadh” OR “Jeddah” OR “Al Ahsa” OR “Makkah”]

The electronic search protocol was applied comprehensively across full-text records to maximize the capture rate of eligible literature.

### 2.3. Eligibility Criteria and Study Selection

#### 2.3.1. Eligibility Criteria

Studies were eligible for inclusion if they met the following predefined criteria:

- **Population:** Studies had to include adults, older adults (aged 50 years or older), healthcare workers, or the general public residing within Saudi Arabia. Studies focusing exclusively on pediatric populations or populations residing outside of Saudi Arabia were excluded.
- **Concept:** Studies had to have conducted a quantitative assessment of knowledge, awareness, attitudes, perceptions, acceptance, hesitancy, barriers, facilitators, or clinical utilization regarding herpes zoster (HZ) or the herpes zoster vaccine (HZV). Studies that did not explicitly address HZ or the HZV and purely qualitative designs lacking measurable, discrete outcomes were excluded.
- **Context:** Studies had to be geographically restricted to the Kingdom of Saudi Arabia. Any studies conducted outside of Saudi Arabia, including those from neighboring regional Arab or Gulf nations, were excluded.
- **Study Types:** Eligible designs comprised peer-reviewed cross-sectional studies, cohort designs, retrospective analyses, surveys, and formal study protocols. Studies evaluating theoretical behavioral constructs rather than descriptive baseline data, gray literature, conference abstracts, dissertation theses, editorials, and commentaries were excluded.
- **Language and Period:** Studies were restricted to English-language studies published between January 2022 and January 2026. Publications printed prior to this period or written in other languages were excluded.

#### 2.3.2. Study Selection Process

The screening process was carried out in two sequential stages using Rayyan web platform (Rayyan Systems Inc., Cambridge, MA, USA, 2026), originally developed by the Qatar Computing Research Institute [23] to facilitate an organized, independent review. To ensure complete methodological transparency, the software was utilized strictly as an administrative data repository to manage titles/abstracts, remove duplicates, and track manually applied inclusion/exclusion labels; no autonomous AI screening algorithms, predictive machine-learning exclusions, or automated screening models were employed at any stage.

The screening and selection process was conducted entirely by the author. To eliminate potential selection bias and safeguard data extraction integrity, a highly structured, pre-tested data-charting framework was strictly followed, and a sequential double-screening cycle was performed by the author two weeks apart to mathematically verify and maintain high intra-rater consistency.

Title and Abstract Screening: The author evaluated all unique titles and abstracts against the predefined eligibility criteria to assess initial clinical relevance.

Full-Text Evaluation: Full-text articles of all potentially eligible records were retrieved and subjected to a rigorous, independent review by the author to definitively confirm final inclusion.

To minimize missing literature, the reference lists of all included articles were manually hand-searched to capture additional eligible studies.

**Figure 1:**
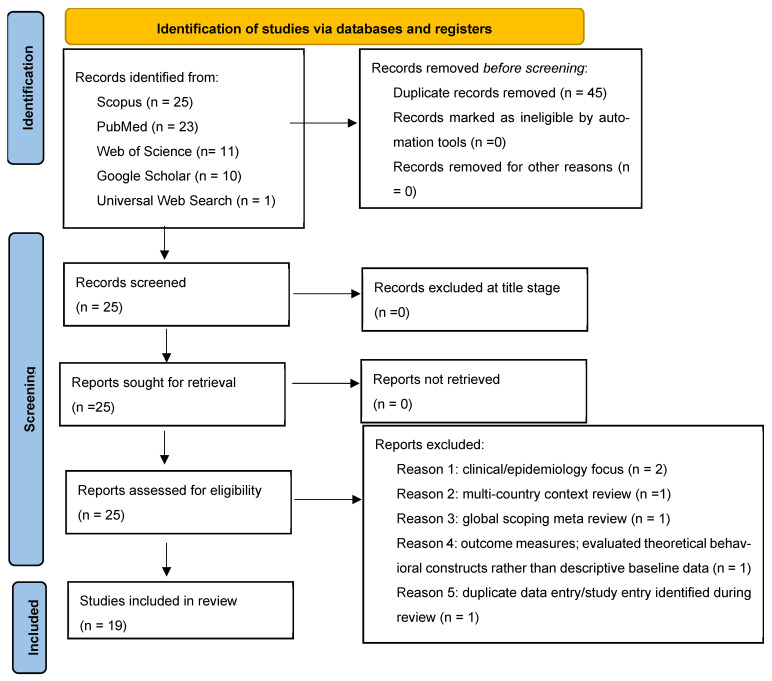
The complete screening and selection workflow visually mapped in a PRISMA flow diagram.

In Figure 1, the screening and selection workflow is visually mapped in a PRISMA flow diagram. Source: Page MJ, et al. BMJ 2021;372:n71. doi: 10.1136/bmj.n71. This work is licensed under CC BY 4.0. To view a copy of this license, visit https://creativecommons.org/licenses/by/4.0/ (accessed on 22 May 2026).

### 2.4. Data Charting and Analysis

#### 2.4.1. Data Extraction Strategy

Data were extracted using a structured charting form developed a priori and iteratively refined by the author to capture variables aligned with the study objectives. The author extracted data from each included paper, and they were subsequently cross-checked for accuracy.

The standardized form systematically captured core variables from each unique cohort study, which included the following:

- **Author and Year:** The full bibliographic reference details, identifying the primary author and year of publication.
- **Geographic Region:** The specific administrative region or city within Saudi Arabia where data collection occurred, such as Riyadh, the western region, or a national sample.
- **Study Design and Setting:** The methodological framework used alongside the setting, including community settings, primary healthcare centers, hospitals, public online spaces, or national datasets.
- **Population and Sample Size:** Target demographic characteristics, such as age ranges and patient versus healthcare worker status, along with the total participant sample size.
- **Measures Assessed:** The specific primary metrics evaluated, such as vaccine hesitancy scales, general knowledge scores, or awareness parameters.
- **Key Findings:** The principal statistical and descriptive results regarding disease awareness, vaccine perception, and actual uptake.
- **Barriers and Facilitators:** Structural, financial, or psychological obstacles to vaccination (such as safety concerns or lack of awareness) alongside positive drivers (such as explicit physician recommendations).
- **Recommendations:** Actionable strategies proposed by the authors to enhance public awareness and clinical implementation of the vaccine.

To ensure rigorous data extraction integrity within a single-reviewer framework, a highly structured, pre-tested data charting form was strictly followed to prevent subjective variance. Additionally, a sequential double-screening round of the compiled literature was performed by the author two weeks apart to mathematically verify and maintain high intra-rater consistency during the data extraction phase.

#### 2.4.2. Analytical Framework

The analytical approach utilized a dual methodology combining descriptive numerical summaries with an inductive thematic synthesis to map the landscape of HZV research in Saudi Arabia.

Descriptive statistics were used to quantify the distribution of literature across geographic regions, chronological publication years, study designs, and sample sizes, offering a macro-level overview of the current research landscape. In parallel, a thematic analysis was conducted to uncover recurring patterns, common behavioral determinants, and socio-demographic influences affecting vaccine uptake and hesitancy. Themes were generated inductively directly from the extracted text and refined.

#### 2.4.3. Data Mapping and Outcome Categorization

To evaluate the presence of an intention–behavior gap in the existing literature, the included studies were systematically audited and categorized based on their primary outcome measurements. The studies were classified into two distinct groups: (1) those measuring direct uptake or practices (defined as the empirical tracking of real-world vaccine deployment via verified clinical records, self-reported vaccination status, or institutional administration metrics) and (2) those measuring intent or willingness only (defined as the assessment of hypothetical vaccine acceptance, stated eagerness to be vaccinated based on future provider advice, or general vaccine hesitancy scales). The distribution of these outcome metrics across the final study pool was tabulated to provide an empirical basis for analyzing the discrepancy between theoretical acceptance and actual clinical behavior.

### 2.5. Ethical Considerations

As this scoping review relied solely on publicly available, peer-reviewed literature and involved no direct contact with human subjects, formal institutional ethical review and approval were not required.

### 2.6. Quality Appraisal

Consistent with the PRISMA-ScR guidelines, no formal methodological quality assessment was conducted, as the purpose of a scoping review is to map the breadth of available evidence rather than evaluate study quality.

## 3. Results

### 3.1. Results of the Literature Search (PRISMA Text)

The initial search across the databases yielded 69 raw records (PubMed: *n* = 23; Scopus: *n* = 25; Web of Science: *n* = 11; Google Scholar: *n* = 10). Following the manual removal of 45 duplicate entries, 24 unique articles advanced to the screening phase. Manual reference-tracking and hand-searching identified 1 additional eligible study by Alfandi et al. [12] resulting in 25 records for comprehensive title and abstract screening. No records were excluded during the initial title and abstract phase (*n* = 0); thus, all 25 reports were successfully retrieved for full-text eligibility assessment. During the final critical appraisal phase, During the final critical appraisal phase, five articles were excluded based on strict criteria: two focused strictly on clinical/epidemiological profiles rather than vaccine behavior; one presented a broad, multi-country regional policy review; one was a global scoping meta-review lacking independent, localized raw data; and one evaluated theoretical behavioral constructs rather than descriptive baseline data and one was identified as duplicate during peer review. Ultimately, 19 unique primary studies conducted exclusively in the Kingdom of Saudi Arabia (KSA) fulfilled all inclusion criteria and were included in the final qualitative synthesis.

### 3.2. Methodological and Geographic Profiles

The 19 included studies were published between April 2022 and January 2026, collectively representing a combined sample size of over 12,000 participants. The baseline research landscape shows a critical chronological pivot following the Saudi Ministry of Health (MOH) official launch of the herpes zoster vaccine (HZV) on 29 September 2022. Early studies focused on broad, non-specific vaccine hesitancy metrics, whereas later studies focused explicitly on HZV-specific implementation parameters.

**Methodological Profiles and Data Extraction of Included Studies.** Table 1 outlines the foundational methodological characteristics and baseline metrics of the reviewed literature. The overall map base relies heavily on cross-sectional designs across the Kingdom of Saudi Arabia.

Table 2 presents a structured thematic analysis of the individual, interpersonal, and factors affecting herpes zoster vaccine (HZV) uptake in Saudi Arabia. A critical cross-cutting paradox characterizes the compiled literature: while latent public intent and general attitudes toward vaccination are favorable, actual uptake faces deep-seated cognitive and logistical barriers. The primary barrier identified across the majority of the studies is a profound deficit in public awareness, with most eligible adults completely unaware that a zoster immunization program is offered locally. This is compounded by extensive knowledge gaps regarding the clinical severity of post-herpetic neuralgia (PHN), which inadvertently foster a false sense of immunity and diminish perceived personal vulnerability among eligible middle-aged populations.

On a psychological level, deep-rooted concerns regarding vaccine reactogenicity and post-injection adverse effects serve as major barriers frequently aggravated by digital misinformation networks. The analysis reveals institutional and provider-level structural deficits. Leading among these is an extensive lack of proactive physician training during routine adult wellness visits. This system-level omission is further exacerbated by the absence of structured continuing medical education (CME) pathways for frontline clinicians and a lack of occupational or institutional vaccination mandates.

Table 3 provides a comprehensive, stratified overview of the behavioral and clinical trends mapped across six core functional domains: knowledge, awareness, attitudes, acceptance/willingness, actual uptake, and hesitancy. The baseline knowledge and awareness domains are consistently characterized in the literature as low to moderate. This deficit is primarily driven by widespread public confusion regarding the specialized two-dose zoster vaccine (HZV) schedule, alongside an overarching lack of clarity regarding the local target age eligibility criteria (≥50 years). Conversely, baseline attitudes and acceptance/willingness demonstrate a highly favorable trajectory, trending neutral to positive. This indicates a robust, latent public readiness to receive the vaccine, provided that a trusted healthcare professional initiates the clinical counseling process. However, this receptive mindset fails to materialize into proactive health-seeking behavior. A profound “intention–behavior gap” separates hypothetical willingness from clinical execution, leaving actual uptake rates critically low—frequently hovering below 10% across almost all sampled cohorts, including high-risk groups. This behavioral stagnation is actively sustained by vaccine hesitancy, the levels of which remain moderate to high. Rather than stemming from a systemic anti-vaccine sentiment, this hesitancy is primarily driven by concerns regarding short-term injection site reactogenicity, which are continuously amplified by local digital and social media echo chambers.

Table 4 maps the behavioral metrics across the geographic landscape of Saudi Arabia, identifying distinct regional variations. Riyadh and the central regions demonstrate moderate baseline awareness profiles, supported by a dense concentration of tertiary academic medical centers and active primary healthcare (PHC) networks. Al-Ahsa and the Eastern Province demonstrate elevated public awareness metrics due to the historical deployment of local, targeted public health education projects; however, evaluations of older adults in the region show that 85.4% remain unvaccinated due to low-risk perception. Conversely, the western region, Jazan Province, and the northern border/Al-Jouf regions display the most acute knowledge deficits. In the remote northern cohorts, over half of the public reports poor baseline knowledge, and community members rely heavily on the internet or social circles rather than medical professionals for vaccine information. Table 4 shows the geographic variations in and regional analysis of the findings.

Table 5 provides a direct comparative analysis between the general population [GP] and healthcare workers [HCWs]. Medical professionals and family medicine residents demonstrate a significantly higher baseline awareness of HZ and more highly supportive public health attitudes toward adult immunization campaigns than the general public. However, detailed clinical validation studies reveal critical vulnerabilities among providers: training residents and staff show clear knowledge gaps regarding updated national adult immunization schedules and specific patient eligibility criteria. Furthermore, the HCWs themselves exhibit low personal vaccination rates, revealing a parallel gap between theoretical knowledge and personal preventative practice. Frontline professionals mention an absence of clear workplace vaccination policies and localized supply chain or stocking instabilities as their primary barriers to active patient counseling.

Table 6 illustrates a clear structural imbalance in the literature between theoretical metrics and practical execution. A total of 11 studies (60%) exclusively evaluated intent/willingness only, examining how participants state that they would behave in hypothetical scenarios. These papers consistently report high baseline interest and a strong hypothetical willingness to receive the herpes zoster vaccine (HZV), particularly if explicitly recommended by a healthcare provider. Conversely, only eight studies (40%) captured direct clinical uptake/practices by tracking actual vaccine receipt or verified institutional administration records. Across these eight behavior-focused studies, actual vaccine coverage was critically low, consistently remaining below 10% to 12%. This division outlines the empirical foundation of the regional intention–behavior gap, demonstrating that strong public intent and highly positive theoretical willingness do not successfully translate into actual clinical vaccination rates.

## 4. Discussion

This scoping review synthesizes empirical evidence from 19 cross-sectional studies conducted in the Kingdom of Saudi Arabia (KSA), framing the modern landscape of herpes zoster (HZ) and herpes zoster vaccine (HZV) implementation. Methodologically, this synthesis strictly adheres to the advanced frameworks established by Arksey and O’Malley [21] and Levac et al. [22], utilizing the updated Joanna Briggs Institute (JBI) methodological guidance [19], and it is reported according to the PRISMA Extension for Scoping Reviews (PRISMA-ScR) protocol [20].

The empirical landscape outlined in this review identifies a critical historical shift following the official announcement by the Saudi Press Agency and the Ministry of Health (MOH) on 29 September 2022, which introduced the HZV at no cost across primary healthcare centers [8]. While earlier regional works, such as the cohort study by Binsaeedu et al. [5], primarily evaluated retrospective epidemiological incidence data to establish disease burden, post-2022 data reveal an acute operational paradox. Despite the total elimination of financial barriers, actual clinical uptake remains severely low, at less than 10%. This low rate reflects the modern global struggle against vaccine hesitancy, which the World Health Organization classifies as a top threat to planetary health [3].

### 4.1. The Knowledge–Awareness Deficit: A Localized and Global Phenomenon

The stagnant HZV uptake rate below 10% across the Kingdom of Saudi Arabia (KSA) exposes a stark operational paradox following the Ministry of Health’s free vaccine rollout on 29 September 2022 [5,8,15]. Local data show this implementation gap is driven by a profound cognitive blind spot regarding disease severity rather than complete ignorance of the virus itself. While superficial familiarity with ‘shingles’ as a basic skin rash is high across the Western, Qassim, and Aseer regions [15,16,17], public understanding of post-herpetic neuralgia (PHN) is critically restricted [6,12,13]. When patients minimize HZ as a transient dermatological concern, their low perceived personal risk prompts them to ignore prophylaxis, matching longitudinal global trends where low risk perception directly stalls adult vaccination [1,2]. However, while Western nations successfully mitigated this during initial rollouts through centralized public education media campaigns [27,29], KSA’s informational ecosystem remains highly fragmented and uniquely vulnerable to unverified digital misinformation channels that magnify anxieties regarding post-injection side effects [4,7,24]

### 4.2. High-Risk Cohorts and the Intention–Behavior Paradox

This risk-perception gap creates a severe intention–behavior paradox among high-risk subpopulations, such as adults living with type 2 diabetes. While diabetic cohorts in Riyadh and Aseer express highly positive theoretical willingness to receive the vaccine, their actual empirical utilization completely stalls in the absence of direct clinician intervention [9,17]. This reliance on passive patient initiation represents a known systemic failure documented across European and North American adult vaccination frameworks [30]. The data from the Aseer region must be interpreted with caution, as they were derived exclusively from high-risk clinical cohorts with uncharacteristically high health-seeking behavior, rather than community-wide sampling [4]. This specific cohort dynamic narrows the intention-behavior gap artificially compared to the healthy general public. Systemically, when public health strategies rely on passive patient request rather than active clinical triggers, vulnerable populations remain unprotected, a reality heavily exacerbated in peripheral zones like Jazan Province where rural older adults face severe structural knowledge deficits [24].

### 4.3. The Systemic Breakdown: Provider Knowledge and Institutional Gaps

These patient-level deficits are profoundly worsened by unexpected knowledge gaps and institutional bottlenecks among frontline healthcare workers (HCWs). Local evaluations challenge the assumption that providers possess complete operational expertise; family medicine residents and primary care physicians exhibit critical clinical errors regarding the HZV protocol, target age cutoffs, and the specific two-dose sequence timeline [14,18]. This provider-level uncertainty directly explains the low personal vaccine uptake among eligible HCWs themselves [15]. This deficiency aligns with global whitepapers confirming that medical curricula heavily prioritize pediatric immunization while neglecting adult guidelines [31,32]. In Saudi clinics, these cognitive provider gaps are compounded by structural barriers, including severe time constraints, missing institutional mandates, a lack of continuing medical education (CME) modules, and inconsistent regional supply chains [10,12]. When clinicians are structurally unsupported or uncertain about dosing intervals, they routinely omit vaccine advocacy during routine patient consultations

### 4.4. Clinical Activation as the Definitive Catalyst

Despite geographic and demographic variances across the provinces, the pooled data converge on a singular structural solution: shifting from passive vaccine availability to active clinical activation. Across all regional data—from metropolitan hubs like Riyadh and Jeddah to peripheral administrative zones like Al-Ahsa and Jazan—a direct, proactive physician recommendation was statistically validated as the single strongest predictor of vaccine acceptance and uptake [6,9,11,12,24,25]. When a trusted primary care clinician actively initiates the discussion, addresses PHN pain profiles, and reassures the patient regarding reactogenicity, public hesitancy decreases dramatically [16,27,28,33]. 

### 4.5. Limitations

This scoping review has certain limitations.

First, this scoping review is limited by its single-reviewer design, which increases the individual selection bias and data extraction errors compared to the standard dual-reviewer frameworks mandated by JBI protocols. To mitigate this risk and safeguard data integrity, rigorous quality-control protocols were enforced: data extraction was strictly driven by a highly structured, pre-tested charting framework to minimize subjective drift, and a sequential double-screening round was executed by the author two weeks apart to verify and maintain high intra-rater consistency.

Second, the included body of evidence relies exclusively on cross-sectional survey designs, which capture data endpoints at a single point in time and are incapable of establishing direct causal relationships.

Third, a significant portion of the compiled literature utilizes online convenience sampling methods via social media channels. This recruitment method introduces distinct selection biases, over-representing younger, more digitally literate, and highly educated individuals while systematically under-sampling isolated, elderly, or rural populations who face higher clinical risks.

Fourth, because multiple included surveys were executed independently within identical large metropolitan hubs (specifically Riyadh and Al-Ahsa) during overlapping timelines, the risk of minor sample duplication or population double-counting across separate publications cannot be entirely ruled out.

Fifth, the synthesized literature relies on self-reported survey data regarding vaccination history rather than verified electronic health or medical records. This dependency on subjective recall introduces potential social desirability bias and may obscure the precise mathematical evaluation of the true intention–behavior gap.

## 5. Conclusions and Recommendations

### 5.1. Conclusions

This scoping review establishes that the shingles vaccine is now distributed completely free across Saudi Arabia (HZV) and is associated with high public intent (70%), it did not automatically translate into clinical compliance, leaving actual uptake stagnant below 10%. The compiled data conclusively pinpoints a critical knowledge-risk deficit: the public routinely conceptualizes herpes zoster as a transient dermatological issue rather than a debilitating neurological threat, directly driving low personal risk perception. Furthermore, the review identifies a crucial systemic breakdown at the provider level, characterized by actionable gaps in multi-dose scheduling expertise among frontline clinicians and a lack of active electronic tracking systems. Ultimately, the empirical evidence proves that adult vaccine uptake in the Kingdom is not self-initiated; rather, a proactive, direct clinician recommendation remains the single most definitive statistical predictor of patient activation and vaccine acceptance.

### 5.2. Recommendations

To bridge the gap between vaccination intent and actual vaccine uptake, public health programs should focus on three clear actions:

Use Automatic Clinic Alerts: Automatic pop-up reminders should be incorporated into electronic health records (EHRs) to flag patients aged over 50 years during routine check-ups. For smaller clinics without advanced computers, checkboxes should be added to standard nursing intake forms.

Train Frontline Doctors: Quick, mandatory training modules should be developed for family medicine doctors and residents to clear up confusion regarding the two-dose timeline, target age rules, and common side effects.

Change the Public Message: In public media, shingles should not be framed as simply a skin rash. Public health campaigns must focus on the severe pain caused by nerve damage (PHN), reassure the public about vaccine safety, and clearly state that the HZV is completely free at all primary healthcare centers.

## Figures and Tables

**Table 1:** Detailed summary of the 19 included studies conducted in Saudi Arabia.

Citation [Ref #]	Region in KSA	Population and Setting	Sample Size [*n*]	Study Design	Core Objective	Key Findings	Barriers/Facilitators
**Alaamri et al.** [4]	National	General population	1500	Cross-sectional	General vaccine hesitancy	High baseline vaccine hesitancy noted	Barriers: Misinformation and deep-rooted cultural beliefs
**AlMuammar et al.** [6]	National	General adults	1010	Cross-sectional	HZV awareness and acceptance	Low baseline awareness; moderate acceptance	Facilitator: Strong physician recommendation improves uptake
**Alrufayyiq et al.** [7]	Riyadh	General population	385	Cross-sectional	Assess KAP toward HZ vaccination	Low knowledge (31%), low awareness (26%), very low uptake (5%)	Barriers: no physician recommendation (79%), fear of side effects (34%)
**AlSaif et al.** [9]	Riyadh	Chronic diseases	780	Cross-sectional	Patient willingness and trends	Favorable attitudes; low clinical execution/practice	Barrier: Acute lack of clear local clinical guidelines
**Bohamad et al.** [10]	Al Ahsa	General population	600	Cross-sectional	Knowledge and vaccine acceptance	Poor knowledge; acceptance rates improve with target education	Barrier: Complete lack of public health information
**Algarni et al.** [11]	Jeddah	General population	800	Cross-sectional	Public perceptions	Common clinical misconceptions regarding virus severity	Immediate need for community-level public education
**Alfandi et al.** [12]	Al Ahsa	General population	700	Cross-sectional	KAP toward HZV	Low clinical knowledge but highly positive baseline attitudes	High need for structured institutional awareness campaigns
**Alatawi et al.** [13]	Tabuk Region, Northwestern Saudi Arabia	Adults more than 18	446	Cross-sectional	Assess awareness, attitudes, acceptance of HZ vaccine	vaccine knowledge 48%, uptake 5.7%, willingness 82.5%	Barrier: misconceptions, low perceived risk
**Al-Hazmi, A.** [9]	Riyadh	Adults aged age 50 years with chronic diseases; recruited from outpatient clinics of a tertiary hospital.	333	Cross-sectional	Assess RZV vaccine uptake, willingness, and behavioral determinants	Low uptake (12%); moderate willingness (45.7%); overall low baseline knowledge.	**Barrier:** Low perceived disease risk. **Facilitator:** Physician recommendation and higher knowledge.
**Moustafa et al.** [14]	National	Healthcare workers	1200	Cross-sectional	HCW knowledge and practice	Moderate knowledge; surprisingly low personal vaccination rates	Barrier: Total lack of dedicated workplace training
**Zeried et al.** [15]	National	General population	939	Cross-sectional	KAP toward HZV	Low awareness; high knowledge	Barrier: misleading internet source as 24.1% rely on it
**Alolayan et al.** [16]	Qassim Region	General adults [age 50]	439	Cross-sectional	Knowledge and willingness to receive HZV	57.9% had poor knowledge; only 22.8% vaccinated	Barrier: Limited knowledge distribution via public channels
**Alamri et al.** [17]	Aseer Region	Patients with DM	350	Cross-sectional	Access barriers	Safety concerns; prominent lack of clear data sheets	Barrier: High need for explicit clinical reassurance
**Alahmari et al.** [18]	Riyadh	Family medicine residents	154	Cross-sectional	Resident knowledge and practice attitudes	96.8% report vaccine availability but reveal timing gaps	Barrier: Lack of targeted primary care immunization education
**Alhazmi et al.** [24]	Jazan Province	Older adults	300	Cross-sectional	Baseline knowledge	Deficient baseline knowledge across all age cohorts	Strong requirement for structural institutional campaigns
**Al-Metwali et al.** [25]	Qassim region	Patients with DM	410	Cross-sectional	Assess the acceptability of the vaccine	25% of the diabetic patients were initially ready to accept the HZ vaccine on their own	A low baseline optimization of personal susceptibility and a diminished perceived threat of contracting acute HZ infections inhibited proactive health-seeking behaviors
**Gouda et al.** [26]	Northern Border Region	General adults	708	Cross-sectional	Attitudes toward vaccination and vaccination practices	low knowledge, 60.7% had a positive attitude, 10% showed good practice	Facilitator: Free vaccine availability
**Aldhawyan et al.** [27]	Eastern Province	Older adults [age 50]	431	Cross-sectional	Awareness and uptake insights	High HZ awareness [86.5%] but 85.4% remain unvaccinated	Barriers: Fears of side effects and low risk perception
**Almalki et al. (2024).** [28]	Central Region of Saudi Arabia	General public	385	Cross-sectional	Public KAP evaluation	Low knowledge, positive attitude, low vaccination practice	Lack of awareness, no physician recommendation, misconceptions/Physician advice, education, belief in effectiveness

**Table 2:** Thematic analysis of barriers to and facilitators of HZV uptake.

Category	Specific Barrier/Facilitator	Number of Studies Reporting (*n* = 19)	Key Narrative/Implication
Lack of Awareness	Low awareness of HZ and HZV	1, 4, 5, 6, 9, 10, 11, 12, 14, 18, 15, 19	A highly prevalent barrier across the literature; a clear majority of sampled populations are unaware that a dedicated vaccine is offered locally.
Lack of Awareness	Poor understanding of PHN	10, 11, 16,	Documented in a subset of studies; a lack of knowledge regarding post-herpetic neuralgia complications reduces perceived clinical severity among those surveyed.
Knowledge Gaps	Misconceptions about severity	3, 14,	An emerging finding in few studies; indicates a localized public belief that herpes zoster is either highly rare or clinically mild.
Knowledge Gaps	Confusion about schedule/age	1, 4, 5, 15, 16	Noted in specific cohorts; target populations show uncertainty regarding regional cutoff ages (age of 50 years or more), and some providers show dosing gaps.
Vaccine Hesitancy	Safety and side effect concerns	3, 9, 11, 18,	Frequently noted fear of post-injection reactogenicity and side effects across several surveyed populations.
Vaccine Hesitancy	Low perceived susceptibility	3, 10, 16,	Observed among certain studies, where healthy older adults assume that they are not at risk because they are currently “in good health.”
Healthcare System	Lack of physician initiation	1, 2, 5, 13	Identified in specific health system contexts; while a strong catalyst for uptake, a direct provider recommendation is reportedly not delivered routinely in these settings.
Healthcare System	Guidelines and stocking deficits	15, 16	A localized organizational barrier was noted in a few studies; specific primary health settings report inconsistent vaccine stocking or a lack of clear local guidelines.
Facilitators	Strong provider recommendation	1, 2, 5, 13	Consistently highlighted in relevant studies as an impactful clinical catalyst to close the intention–behavior gap.
Facilitators	Targeted public health campaigns	4, 6, 7, 10, 12, 14, 17	Supported by a substantial number of studies; direct exposure to organized print, digital, or primary care counseling platforms significantly reverses hesitancy.

**Table 3:** Overall synthesis by functional domain knowledge, awareness, attitudes, acceptance/willingness, actual uptake, and hesitancy.

Domain	Overall Trend	Key Synthesis of Findings
Knowledge and Awareness	Low to Moderate	Limited understanding of PHN severity and widespread confusion about the two-dose HZV schedule. Healthcare workers (HCWs) display better baseline knowledge but demonstrate gaps regarding official national deployment guidelines.
Attitudes and Acceptance	Neutral to Positive	High latent willingness to vaccinate across cohorts *if* directly recommended by a primary care physician. Intent to accept improves exponentially following brief educational or digital media interventions.
Actual Uptake	Critically Low	A profound “intention–behavior gap” exists. While hypothetical willingness is high, actual clinical coverage consistently hovers below 10% across almost all sampled cohorts, including high-risk diabetic patients and frontline HCWs.
Hesitancy Drivers	Moderate to High	Hesitancy is actively fueled by unverified medical misinformation circulating through local social media networks, paired with acute fears of short-term reactogenicity.

**Table 4:** Geographic variations in and regional analysis of the findings.

Region	Studies Conducted	Knowledge Level	Awareness Level	Attitudes Toward HZV	Acceptance/Uptake	Key Regional Notes
Riyadh (general adults and family medicine residents)	4, 14	Moderate	Moderate	Neutral to positive	Low uptake [~12%]	Higher localized awareness due to a heavy concentration of tertiary academic medical centers and active primary healthcare [PHC] networks. Frontline family medicine training residents show high disease awareness but notable clinical schedule/timing gaps.
Al-Ahsa and Eastern Province (general public and older adults)	5, 7, 18	Moderate	Higher than other regions	Positive	Moderate willingness; low actual uptake	This region demonstrates strong general awareness of the virus itself, heavily driven by targeted public health projects. However, recent data among older adults [Study 15] reveal that up to 85.4% remain clinically unvaccinated due to low-risk perception and side effect concerns.
Western region (general population): Jeddah, Makkah, Madinah	6, 11	Low	Low	Neutral	Low acceptance and uptake	High population diversity; reports point to inconsistent structural access to adult-specific vaccination programs and pervasive public misconceptions regarding shingles severity.
Qassim (general population and older adults)	3, 12	Low	Low	Neutral	Low acceptance and uptake	Local public perception is heavily influenced by online misinformation networks paired with high baseline vaccine hesitancy. Recent evaluations of older adults show that 57.9% have completely poor baseline knowledge [Study 12].
Aseer region (patients with diabetes mellitus only)	13	High	High	Highly positive	High willingness; low actual uptake [21.5%]	Focused specifically on high-risk cohorts [patients with diabetes mellitus], showing an intense “intention–behavior gap”: patients maintain positive efficacy perceptions, yet actual clinical deployment lags due to an acute need for explicit provider reassurance.
Jazan Province (older adults)	15	Very low	Very low	Neutral	Very low uptake	Highlights severe regional knowledge deficits across older adult cohorts; clear indicators show a step-down in awareness due to the absence of active physician–patient communication outside major metropolitan hubs.
National level	1, 2, 8, 9, 10, 16, 17	Low to moderate	Low	Neutral to positive	Critically low uptake [<10%]	Macro-level national patterns reveal a pervasive baseline deficit in public awareness, surprisingly low personal uptake among healthcare workers [HCWs] despite theoretical awareness, and highly suboptimal institutional screening rates.

Note: Findings for the Aseer region are derived exclusively from a high-risk diabetic patient cohort and are not generalizable to the broader, healthy adult population of that region.

**Table 5:** Comparative analysis: general population vs. healthcare workers.

Domain	General Population [GP] Findings	Healthcare Worker [HCW] Findings	Studies Reporting This (*n* = 19)
**Knowledge**	Low to moderate: The general population shows a widespread deficit in knowledge regarding long-term post-herpetic neuralgia [PHN] complications, age criteria, and the two-dose regimen.	Moderate: HCWs show significantly better baseline knowledge than the public, but distinct gaps persist among training residents and staff regarding official national guidelines and scheduling.	GP: 1, 3, 4, 5, 6, 7, 11, 12, 13, 15,18, 19, HCW: 2, 8, 14
**Awareness**	Low: Fewer than half [<50%] of polled adults are independently aware that a specific shingles vaccine is offered locally.	Moderate to high: Most HCWs are fully aware of the HZV, but they frequently lack specific clinical details on recent update policies or local primary care guidelines.	GP: 1, 5, 6, 7, 10, 11, 12, 13, 15,18, 19, HCW: 2, 8, 14
**Attitudes**	Neutral to positive: The general public shows a strong latent intent to accept vaccination if directly advised by a clinician but is heavily held back by safety fears.	Highly positive: HCWs display robust support for adult immunizations as standard preventive medicine and demonstrate clinical willingness to advocate for patients.	GP: 5, 7, 10, 11, 12, 19 HCW: 2, 8
**Acceptance/Willingness**	Moderate: In the general public, acceptance/willingness improves exponentially immediately following localized, basic educational or digital/social media briefings.	Moderate to high: HCWs show extremely high verbal willingness to recommend or receive the vaccine, but this is not reliably translated into proactive clinical practice.	GP: 1, 7, 11, 16, 17 HCW: 2, 8
**Actual Uptake**	Very low [typically <10%]: The vast majority of the eligible population aged 50 years or over and specific high-risk adults such as those with diabetes have never received the HZV.	Very low [<10%]: HCWs show surprisingly low personal clinical vaccination rates despite their superior underlying medical training and everyday healthcare exposure.	GP: 1, 13, 15,18, 19, HCW: 2, 8, 14
**Hesitancy**	Moderate to high: In the general population, hesitancy is aggressively fueled by negative social media echo chambers, general vaccine skepticism, low self-risk perception, and side effect concerns.	Low to moderate: HCWs show lower overall skepticism, but hesitancy persists due to the absence of clear workplace directives or personal safety concerns regarding adverse events.	GP: 1, 3, 5, 6, 10, 13, 15 HCW: 2, 8
**Barriers**	* Absolute lack of public health dissemination. * Safety and post-injection side effect concerns. * Low perceived personal health risk [“in good health”]. * Commercial cost and lack of absolute insurance coverage.	* Absence of clear institutional workplace vaccination policies. * Lack of structured, dedicated primary care CME training modules. * Inconsistent clinical vaccine stocking and regional supply chain blocks.	GP: 1, 3, 4, 5, 6, 7, 10, 11, 12, 13, 15, 18, 19, HCW: 2, 8, 14
**Facilitators**	* Direct, proactive physician recommendation during routine check-ups. * Organized primary care public health campaigns. * Advanced individual educational or socioeconomic attainment.	* Mandatory institutional clinical training modules. * Clear integration of adult vaccine checkpoints at primary care triage desks. * Readily available institutional guidelines for training residents.	GP: 1, 4, 5, 7, 11, 12, 13, 15,18, 19, HCW: 2, 4, 8

**Table 6:** Outcome metric categorization of included studies (*n* = 19).

Primary Outcome Measured	Number of Studies (*n*)	Study Index References	Key Dynamic Observed
Direct clinical uptake/practices (self-reported receipt or institutional administration rates)	8	2, 4, 5, 8, 13, 14,15, 18	Verified actual coverage is consistently low across these cohorts, rarely exceeding 10–12%, even among healthcare workers and high-risk patients.
Intent/willingness only (hypothetical acceptance, willingness under provider advice, or vaccine hesitancy)	11	1, 3, 6, 7, 9, 10, 11, 12, 16, 17, 19	Hypothetical willingness is consistently moderate to high (often ranging between 45% and 75%), being heavily dependent on hypothetical physician endorsement.

## Data Availability

The original contributions presented in this study are included in the article/Supplementary Materials; further inquiries can be directed to the corresponding author.

## Supplementary Materials

The following supporting information can be downloaded at: https://www.mdpi.com/article/10.3390/vaccines14070565/s1, Supplementary File S1: Search Strategy, Search Strings, and Record Database Yield for Herpes Zoster Vaccine Perceptions and Uptake in Saudi Arabia, PRISMA-ScR-Fillable-Checklist_10Sept2019, PRISMA 2020 flow diagram for new systematic reviews which included searches of databases and registers only.

## Abbreviations

HZV	Zoster Vaccine
EHR	Electronic Health Record
MOH	Ministry of Health
SCFHS	Saudi Commission for Health Specialties
CME	Continuing Medical Education
PHN	Post-Herpetic Neuralgia
PHC	Primary Healthcare
